# A Population-based Study of Hospital Admission Incidence Rate and Bacterial Aetiology of Acute Lower Respiratory Infections in Children Aged Less Than Five Years in Bangladesh

**Published:** 2007-06

**Authors:** Abdullah H. Baqui, Mahbubur Rahman, K. Zaman, Shams El Arifeen, Hafizur Rahman Chowdhury, Nazma Begum, Gaurav Bhattacharya, Rashid A. Chotani, Mohammad Yunus, Mathuram Santosham, Robert E. Black

**Affiliations:** 1 Johns Hopkins University Bloomberg School of Public Heath, 615 N. Wolfe St., Baltimore, MD 21205, USA; 2 ICDDR,B, GPO Box 128, Dhaka 1000, Bangladesh

**Keywords:** Acute lower respiratory infections, Child, Drug resistance, Microbial, Haemophilus influenzae, Hospitalization, Infant, Morbidity, Streptococcus pneumoniae, Bangladesh

## Abstract

The research was carried out to study the rate of population-based hospital admissions due to acute lower respiratory infections (ALRIs) and bacterial aetiology of ALRIs in children aged less than five years in Bangladesh. A cohort of children aged less than five years in a rural surveillance population in Matlab, Bangladesh, was studied for two years. Cases were children admitted to the Matlab Hospital of ICDDR,B with a diagnosis of severe ALRIs. Bacterial aetiology was determined by blood culture. Antimicrobial resistance patterns of Haemophilus influenzae type b (Hib) and Streptococcus pneumoniae (Spn) isolates were determined using the disc-diffusion method. In total, 18,983 children aged less than five years contributed to 24,902 child-years of observation (CYO). The incidence of ALRI-related hospital admissions was 50.2 per 1,000 CYO. The incidences of ALRI were 67% higher in males than in females and were higher in children aged less than two years than in older children. About 34% of the cases received antibiotics prior to hospitalization. Of 840 blood samples cultured, 39.4% grew a bacterial isolate; 11.3% were potential respiratory pathogens, and the rest were considered contaminants. The predominant isolates were Staphylococcus aureus (4.5%). Hib (0.4%) and Spn (0.8%) were rarely isolated; however, resistance of both these pathogens to trimethoprim-sulphamethoxazole was common. The rate of ALRI-related hospitalizations was high. The high rate of contamination, coupled with high background antibiotic use, might have contributed to an underestimation of the burden of Hib and Spn. Future studies should use more sensitive methods and more systematically look for resistance patterns of other pathogens in addition to Hib and Spn.

## INTRODUCTION

More than 10 million children die each year, most from preventable causes and most in poor countries (1). Acute lower respiratory infections (ALRIs) account for about 20% or more than two million of these deaths, making it the leading cause of deaths in children aged less than five years (2,3). Efficacious vaccines against Streptococcus pneumoniae (Spn) and Haemophilus influenzae type b (Hib), the two important causes of ALRI in developing countries, are available (4,5). However, these vaccines are not routinely used in most developing countries because of their high costs and lack of data documenting the burden of the disease. The World Health Organization (WHO) has developed a case-management strategy against ALRIs; this strategy has become the corner stone of national ALRI-control programmes in many countries to decrease mortality due to ALRIs. The strategy is based on diagnosis of ALRIs in children with cough or difficult breathing using easily-discernible signs (tachypnea and chest in-drawing), followed by empiric antimicrobial therapy. The successes of this approach depend on the selection of an antimicrobial agent that is effective against the pathogens most likely to cause fatal ALRIs.

In Bangladesh, about 25% of deaths of children aged less than five years and 40% of deaths in infancy are associated with ALRIs (6). A community-based cohort study in children, aged less than two years, in rural Matlab, observed an annual ALRI incidence of 30/100 child-years (7). A hospital-based study conducted in the Dhaka Hospital of ICDDR,B during 1986-1988 investigated 401 ALRI cases in children aged less than five years for respiratory pathogens. A respiratory pathogen was identified in 30% of patients. The most common pathogen isolated was respiratory syncytial virus (14.4%); Spn and Hib were isolated from 7% and 3% of the cases respectively. The case-fatality rates were 14% due to bacterial pneumonia and 3% due to viral pneumonia (8).

Data on population-based incidence of hospital admissions, aetiology, and antimicrobial resistance are generally scarce and not available from Bangladesh. These data are essential for health-sector planning, including the design of effective case-management strategies and development of effective vaccine policies. This paper presents data on the population-based incidence of hospital admissions due to ALRIs and the bacterial aetiology of ALRIs, particularly the burden of diseases due to Hib and Spn in children aged less than five years in Bangladesh.

## MATERIALS AND METHODS

### Study population, setting, and subjects

The study was carried out at the Matlab field research area of ICDDR,B. Matlab is located about 55 km south-east of Dhaka, the capital of Bangladesh. The field area was originally developed for evaluation of cholera vaccines. To support field studies, a Health and Demographic Surveillance System (HDSS) was established in 1966. Over the years, the system was refined and is currently operational in 142 villages comprising about 210,000 people. The HDSS gathers information on vital events, such as births, deaths, and migrations, on a regular basis through home-visits. Matlab is fairly representative of most parts of rural Bangladesh (8). The surveillance for ALRIs was conducted for two years (July 1999 and June 2001) in children aged less than five years in half of the HDSS area.

### Case definitions

The WHO-recommended case definitions were used for the surveillance (9). Non-severe ALRI was defined as cough or difficult breathing and fast breathing (breathing >60 per minute for infants aged less than two months, >50 per minute for infants aged two months to <1 year, and >40 per minute for children aged 1-4 year(s)) and no chest in-drawing, stridor, or danger signs. Severe ALRI was defined as cough or difficult breathing and any general danger sign or chest in-drawing or stridor in a calm child. The general danger signs were inability to drink or breastfeed, vomiting, convulsions, lethargy, and lack of consciousness.

### Surveillance and case detection

Trained female community health workers (CHWs) —each responsible for about 2,000 persons—made monthly home-visits to improve recognition of illnesses and care-seeking by mothers and families by providing education on signs and symptoms of ALRIs and sources of care. The CHWs were to refer all severe ALRI cases to the Matlab Hospital. During home-visits, the CHWs collected selected data on morbidity and care-seeking for ALRIs.

Many ALRI cases were treated at the community or in peripheral facilities. The study physicians periodically visited the CHWs and peripheral facilities to encourage the providers to refer all severe ALRI cases to the Matlab Hospital and to assess the quality of diagnosis made. ALRI cases referred to the Matlab Hospital were assessed by a physician; only severe ALRI cases were admitted. The hospital was staffed by physicians and had blood culture facilities.

### Laboratory methods

To identify aetiology of ALRIs, blood specimens were collected from admitted cases and were cultured using the standard microbiological methods (10). Briefly, about two mL of venous blood was aseptically drawn and inoculated into 20 mL of trypticase soy broth (TBS) (Oxoid, UK) containing 0.25% sodium polyethanol sulphonate (SPS, Sigma, USA) and incubated at 37 0C for seven days. Blood culture bottles were examined at 14-17 hours and thereafter everyday up to seven days. Subcultures were performed immediately if any turbidity or lysis were observed. Subcultures were performed in blood and chocolate agar plates after 14-17 hours, at 48 hours, and at seven days of incubation, regardless of turbidity or lysis. Blood and chocolate agar plates were made from blood agar base (Oxoid, UK) with 5% sheep blood. Each batch of media was tested for adequate growth of reference strains on the respective media before culturing clinical specimens. Colonies suspected to be H. influenzae were further confirmed on the basis of their growth requirement for hemin and NAD (nicotinamide adenine dinucleotide), using the ‘X’ and ‘V’ factor discs (Difco, USA). Steptococcus pneumoniae and H. influenzae strains were serotyped by the slide agglutination method using type-specific antisera. Antibiograms of the Hib and Spn isolates were performed to determine the antimicrobial resistance patterns against trimethoprim and sulphamethoxazole (co-trimoxazole), ampicillin, chloramphenicol, ceftriaxone, erythromycin, oxacilin, and ciprofloxacin using the disc-diffusion method (11). Results were categorized as sensitive (S), intermediate (I), and resistant (R). The blood cultures were performed in a field laboratory under the direction of the Head of the ALRI Laboratory of ICDDR,B (MR) who provided the initial training on the standarized laboratory methods to the resident microbiologist in the field laboratory and provided regular oversight which included weekly visits to the field laboratory. A 25% sample of blood culture broth and culture plates were transported to the ICDDR,B laboratory in Dhaka for additional plating and characterization.

### Data collection, management, and analysis

Relevant demographic, clinical and epidemiological data were collected from all hospitalized cases using standardized forms and entered onto a standardized computer database with built-in range and consistency checks designed using FoxPro. Age- and sex-specific child-years of observation (CYO) were calculated using HDSS data that provided entry and exit dates of each individual child in the HDSS system. Age- and sex-specific hospitalization rates were calculated per 1,000 CYO. Assuming a Poisson distribution for the number of hospitalizations, 95% confidence intervals and p values were calculated to examine for differences in hospitalization rates with respect to age and sex. Seasonality was depicted by sex and calendar month.

### Ethics

The Research and Ethical Review Committees of ICDDR,B approved the study procedures. Informed consent was obtained from the guardian of each child who participated in the study.

## RESULTS

### Population-based hospitalization rates

In total, 18,983 children aged less than five years contributed to 24,902 CYO, and 1,250 severe ALRI patients were hospitalized, resulting in a hospitalization rate of 50.2 per 1,000 CYO. The rate of hospitalization was about 67% higher in males (62.5; 95% confidence interval [CI] 58.2-67.0) than in females (37.5; 95% CI 34.2-41.1); this difference was statistically significant (p<0.05). Rates of hospitalization were higher for children aged less than two years than in older children. An exception to the high rates in children aged less than two years was a very low rate of severe ALRI observed in neonates (Table 1). The highest rate was observed in the 1-5-month old children [rate per 1,000 CYO (95% CI) 108.9 (95.2-123.9)]. The rate in 6-11-month old children was very similar to that in 1-5-month old children. The ALRI-related admission rates declined with increasing age after the first year of life. About 34% of the cases in this study received antibiotics prior to obtaining blood samples for culture.

**Table 1:** Distribution of population-based hospitalization rates by age-group and sex

Age-group (months)	Male			Female			Total		
	Child-years observed	Hospitalization		Child-years observed	Hospitalization		Child-years observed	Hospitalization	
		No. hospitalized	Rate per 1,000 child-years observed (CD		No. hospitalized	Rate per 1,000 child-years observed CD		No. hospitalized	Rate per 1,000 child-years observed (CD
<1	234	8	34.2 (14.7-67.3)	226	5	22.1 (7.1-51.6)	460	13	28.3† (15.0-48.3)
1-5	1,068	143	133.9∗ (112.8-157.7)	1,026	85	82.8 (66.1-102.4)	2,094	228	108.9† (95.2-123.9)
6-11	1,315	176	133.8∗ (114.7-155.1)	1,244	100	80.4 (65.4-97.7)	2,559	276	107.9† (95.5-121.3)
12-17	1,337	152	113.7∗ (96.3-133.2)	1,278	68	53.2 (41.3-67.4)	2,615	220	84.lt (73.3-96.0)
18-23	1,346	88	65.4∗ (52.4-80.5)	1,298	58	44.7 (33.9-57.7)	2,644	146	55.2† (46.6-64.9)
24-29	1,323	54	40.8 (30.6-53.2)	1,293	40	30.9 (22.1-42.1)	2,616	94	35.9† (29.0-43.9)
30-35	1,287	54	42.0 (31.5-54.7)	1,264	39	30.9 (21.9-42.1)	2,551	93	36.5† (29.4-44.6)
36-41	1,221	54	44.2∗ (33.2-57.7)	1,204	24	19.9 (12.7-29.6)	2,425	78	32.2† (25.4-40.1)
42-47	1,159	29	25.0∗ (16.7-35.9)	1,157	14	12.1 (6.6-20.3)	2,316	43	18.6 (13.4-25.0)
48-53	1,167	15	12.9 (7.1-21.1)	1,158	17	14.7 (8.5-23.5)	2,325	32	13.8 (9.4-19.4)
54-59	1,158	16	13.8 (7.8-22.4)	1,139	11	9.7 (4.8-17.2)	2,297	27	11.8 (7.7-17.1)
Total	12,615	789	62.5∗ (58.2-67.0)	12,287	461	37.5 (34.2-41.1)	24,902	1,250	50.2 (47.4-53.0)

∗Indicates a statistically significant difference (p<0.05) between males and iemales oi the particular age-group; †Indicates a statistically significant difference (p<0.05) between that age-group and age-group ot 54-59 years; CI=Confidence interval

### Seasonality

The rates of population-based ALRI hospitalization showed some seasonal variation. Two peaks were observed in both the study years. The larger peak was observed in the post-monsoon season (November of year 1 and in September of year 2). The smaller peak was observed in the pre-monsoon season (Fig. 1). The seasonal patterns were similar in both males and females, although males consistently had higher rates.

**Fig.**
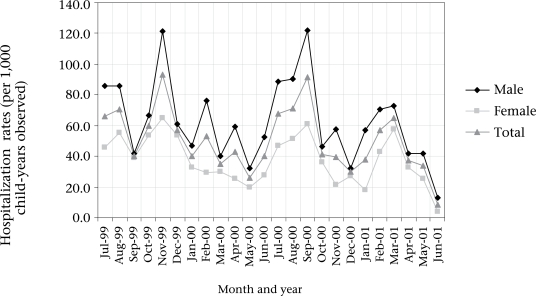
Seasonality of population-based hospitalization rates by sex

### Bacterial aetiology

In total, 840 blood samples were cultured. Bacteria were isolated from 331 (39.4%) specimens, and no bacterial growth was observed in the remaining (60.6%) blood samples. Of the 331 isolates, 95 were considered potential pathogens, and the rest were considered contaminants. The majority (62/95 or 65%) of the pathogenic isolates were identified from children aged 1-11 month(s). The rate of total population-based pathogen isolation per 1,000 CYO was 3.81. The rate of population-based pathogen isolation per 1,000 CYO was highest in <1 month (17.39), followed by 1-5 month(s) (13.37), 6-11 months (10.16), and 12-23 months (3.61) age-groups. The predominant isolates were Staphylococcus aureus (38, 4.5%). Hib and Spn were rarely isolated with only three (0.4%) and seven (0.8%) isolates respectively. Of the three Hib isolates, two were isolated in infancy, whereas four of the seven Spn isolates were isolated in infants aged less than six months. Twenty-eight (3.3%) isolates were gram-negative bacilli (Table 2). Some organisms, such as coagulase negative Staphylococcus (CNS) (140, 16.7%), Neisseria spp. (0.2%), Flavobacterium spp. (0.1%), Micrococcus sp. (6.4%), Viridans streptococci (0.7%), and others (3.9%) were also isolated but were excluded as possible contaminants.

**Table 2:** Aetiology of ALRI in hospitalized children by age-group

Pathogens and contaminants isolated	Age-group (months)						Total (n=840)
	<1 (n∗=58)	1-5 (n=285)	6-11 (n=177)	12-23 (n=218)	24-35 (n=62)	36-59 (n=40)	
Pathogens isolated							
*Haemophilus influenzae* type b	-	1 (0.4)	1 (0.6)	1 (0.5)	-	-	3 (0.4)
*Streptococcus pneumoniae*	1 (1.7)	3 (1.1)	-	2 (0.9)	1 (1-6)	-	7 (0.8)
*Klebsiella* sp.	1 (1-7)	-	2 (1.1)	-	1 (1-6)	-	4 (0.5)
*Pseudomonas* sp.	-	2 (0.7)	3 (1.7)	1 (0.5)	1 (1-6)	1 (2.5)	8 (1.0)
*Escherichia colt*	1 (1-7)	-	-	-	-	1 (2.5)	2 (0.2)
*Acinetobacter* sp.	-	-	1 (0.6)	1 (0.5)	-	1 (2.5)	3 (0.4)
*Salmonella* sp.	-	-	1 (0.6)	-	-	-	1 (0.1)
*Staphylococcus aureus*	5 (8.6)	12 (4.2)	11 (6.2)	5 (2.3)	4 (6.5)	1 (2.5)	38 (4.5)
Gram-negative bacilli	-	9 (3.2)	7 (4.0)	9 (4.2)	2 (3.2)	1 (2.5)	28 (3.3)
*Haemophilus para-influenzae*	-	1 (0.4)	-	-	-	-	1 (0.1)
Total pathogens	8 (13.8)	28 (9.8)	26 (14.7)	19 (8.7)	9 (14.5)	5 (12.5)	95 (11.3)
Population-based pathogen isolation rate (per 1,000 child-years of observation)	17.39	13.37	10.16	3.61	1.74	0.53	3.81
Contaminants isolated							
*Neisseriae* sp.	1 (1-7)	-	1 (0.6)	-	-	-	2 (0.2)
*Flavobacterium* sp.	-	-	1 (0.6)	-	-	-	1 (0.1)
*Viridans streptococci*	-	-	1 (0.6)	4 (1.8)	1 (1-6)	-	6 (0.7)
*Micrococcus* sp.	1 (1-7)	10 (3.5)	15 (8.5)	17 (7.8)	5 (8.1)	6 (15.0)	54 (6.4)
Coagulase-negative *Staphylococcus*	7 (12.1)	31 (10.9)	31 (17.5)	44 (20.2)	14 (22.6)	13 (32.5)	140 (16.7)
Others	4 (6.9)	9 (3.2)	10 (5.7)	5 (2.3)	3 (4.8)	2 (5.0)	33 (3.9)
Total contaminants	13 (22.4)	50 (17.5)	59 (33.3)	70 (32.1)	23 (37.1)	21 (52.5)	236 (28.1)
Total isolates (pathogens and contaminants)	21 (36.2)	78 (27.4)	85 (48.0)	89 (40.8)	32 (51.6)	26 (65.0)	331 (39.4)

Data are numbers (%) of cases; ∗Number of cultures done ALRI=Acute lower respiratory infection

### Antimicrobial resistance

Hib and Spn isolates were tested for antimicrobial resistance. One of the three Hib isolates was resistant to co-trimoxazole, and three of the seven Spn isolates were resistant to co-trimoxazole (Table 3).

**Table 3:** Antimicrobial resistance by pathogen

Pathogen	Microorganisms isolated	Antibiogram done	Antibiotics and sensitivity																							
			Ampicillin				Chloramphenicol				Erythromicin				Oxacillin				Trimethoprim-sulphame-thoxazole				Ceftriaxone			
			S	I	R	N	S	I	R	N	S	I	R	N	S	I	R	N	S	I	R	N	S	I	R	N
*Haemoph Hits influenzae* type b	3	3	3	-	-	-	3	-	-	-	2	-	1	-	-	-	-	3	2	-	1	-	3	-	-	-
*Streptococcus pneumoniae*	7	7	-	-	-	7	7	-	-	-	7	-	-	-	7	-	-	-	4	-	3	-	7	-	-	-

I=Intermediate; N=Not done; R=Resistant; S=Sensitive

## DISCUSSION

Data on rates of population-based hospitalization for ALRIs from developing countries are scarce. In this population-based study of children aged less than five years, the overall rate of hospitalization for ALRIs was about 50.2 per 1,000 CYO. We conducted another population-based study about 10 years ago in three villages of Matlab and observed an annual ALRI incidence of 30/100 CYO (7). The methods of this study were different from the earlier study. In the earlier study, we conducted a twice-weekly surveillance and, therefore, identified respiratory diseases early on and treated the cases presumably averting severe diseases. A Brazilian study reported a hospitalization rate of 2.9% in 1-11-month old children (12); this lower rate could be due to either differences in population characteristics or less-intensive surveillance. The high rates of hospitalization due to ALRIs in our study population are consistent with the cause structure of deaths in children aged less than five year in Bangladesh. A nation-wide verbal autopsy study conducted in Bangladesh attributed 40% of infant deaths and 25% of all deaths of children, aged less than five years, due to ALRIs (6). Since the Matlab Hospital was the only tertiary-care hospital in the study area, it may be assumed that most severe cases seeking care in a hospital were captured by the surveillance. Furthermore, the HDSS may have allowed for a more accurate determination of the number of CYOs.

In our study, the hospitalization rate was very low for neonates, was highest in the postneonatal period (1-11 month(s)), and declined with increasing age after the first year of life. The low rate of hospitalization due to ALRIs in neonates was not expected. The vulnerability of the newborn can be gauged by the fact that approximately two-thirds of deaths of infants and 40% of deaths of children, aged less than five years, occur during this period (13). Despite the increased risk, the neonatal period has remained relatively unattended in developing countries due to several biological, social and economic reasons. The unexpectedly low rate of hospitalization due to ALRIs in neonates could be due to poor recognition of illness or low care-seeking or both. Since the signs and symptoms of infections, including ALRI, is less pronounced in neonates, recognition of illness may be difficult, and infections may progress more quickly allowing less time to seek care (14). The low care-seeking may also be due to sociocultural constraints, including cultural restrictions against seeking care outside home in the first month of life and not giving allopathic medicines to young babies because they are considered too strong (14). This behaviour is encouraged by maternal beliefs about the causes of ALRIs and the fact that indigenous and traditional healers constitute more than 80% of village practitioners in this setting (15,16). Since neonatal mortality remains unacceptably high, interventions need to be designed to reduce neonatal infections and to increase early identification and treatment of sepsis, including ALRI, in the neonatal period.

A statistically significant difference was observed between males and females with the overall hospitalization rate in males being as much as 67% higher than in females. This could be due to a higher incidence of ALRIs in males than in females, as reported in previous studies (17–19). This could also be due to higher rates of care-seeking for male children than for female children, given a strong preference for sons in the South Asian region (20). In 1998, the ALRI-specific mortality in our study area was 14/1,000 and 12/1,000 livebirths in infancy and 1/1,000 and 2/1,000 livebirths in 1-4-year age-group among males and females respectively. The all-cause mortality rates were 45.6 and 55.5/1,000 livebirths in infancy and 4.0 and 5.3/1,000 livebirths in 1-4-year age-group for males and females respectively (8). Although the overall mortality was higher among females than among males, the ALRI-specific mortality among male and female infants was similar. Despite higher care-seeking, similar ALRI mortality in males supports the contention that the incidence of severe ALRI may indeed be higher among males.

There was a noticeable seasonal variation in the rates of hospitalization. Two peaks were observed—one was a larger peak in the post-monsoon and the other one was a smaller peak in the pre-monsoon months. These peaks were observed at about the same time in both the years of the study, although the post-monsoon peak started a little early in 2000 and lasted longer compared to 1999. Studies conducted in this region have observed variations in seasonality. One study observed a winter peak but no summer peak; this study was, however, based on a small sample (7). Other studies have also observed a winter peak but one study reported no seasonal variation (21,22). These differences might be attributable to climatic conditions together with the prevalence of opportunist causative agents that might be predominant in individual conditions. The seasonality patterns were similar in both males and females with females having a consistently lower hospitalization rate through out the study period.

A positive culture was obtained in 331 (39.4%) of the cases. S. aureus (n=38; 4.5%) was the most frequently-found aetiologic agent. A hospital surveillance in Nigerian children reported S. aureus as the major aetiologic agent in 21% of blood samples (23). Characterization of the 3.3% isolates of gram-negative bacilli was not done and could have provided useful aetiologic data. Although coagulase-negative staphylococci (16.7%), Micrococcus sp. (6.4%), Viridans streptococci (0.7%), and other microorganisms (3.9%) were found with varying frequency, they are widely regarded as contaminants and were, therefore, not considered pathogens (24). Other isolates, such as Neisseriae sp., rarely cause ALRI in children, and Flavobacterium sp. are known to cause meningitis in newborns and premature infants or ALRIs in adults with severe underlying illnesses (25–27). Although 1,250 ALRI cases were hospitalized, blood cultures were performed in 840 cases. The loss of 410 potential cases was not desirable. Of the 840 cultures, 236 grew a contaminant. Therefore, only about half of the blood cultures were available for analysis which is a potential limitation of the study. Therefore, the aetilogic findings of this study should be treated with caution. The contaminants might have suppressed the growth of true pathogen. Coagulase-negative staphylococci were the main presumed contaminants in this study and in many other studies in various settings (38–40). As these organisms may occasionally cause serious disease, differentiating bacteraemia from contamination is very important but often difficult (38,39). Coagulase-negative staphylococci-associated bacteraemia usually occurs with implanted foreign devices. One study found that coagulase-negative staphylococci can cause persistent bacteraemia in low-birth-weight neonates (40). Since low birth-weight and undernutrition rates are one of the highest in Bangladesh, sorting out patients with coagulase-negative staphylococci-associated bacteraemia from sample contamination will be important. Strict aseptic measures, strict clinical criteria, and serial blood cultures are most important in differentiating patients with bacteraemia from cases of sample contamination. The growth of coagulase-negative staphylococci in less than 48 hours is significantly associated with bacteraemia (39). Additionally, plasmid profile analysis and phage-typing are useful microbiological tools that should be considered in future studies to distinguish strains causing true bacteraemia from contaminants.

Hib and Spn were rarely isolated in this study. Since ALRIs are characterized by intermittent bacteraemia and low concentrations of bacteria in the blood, blood cultures in ALRI patients underestimate the burden of diseases due to Hib and Spn. To overcome these problems, it has been recommended that blood be obtained for 2-3 separate blood cultures (28). Repeated cultures, however, were not feasible in this study. Moreover, about 34% of the cases in this study received antibiotics prior to obtaining blood samples for culture. The high rate of background antibiotic use coupled with the high rate of contamination might have contributed to the underestimation of the burden of diseases due to Hib and Spn. Alternative approaches are to use the antigen-detection test and lung tap which have much higher sensitivity. Thirty-six to 50% of ALRI cases were diagnosed as bacterial type in some studies in which antigen detection was used for making the diagnosis of Hib and pneumococcal pneumonia (29,30).

The antimicrobial sensitivity results showed that one of the three Hib and three of the seven Spn isolates were resistant to co-trimoxazole which is the drug of choice in the national ALRI-control programme of Bangladesh. Although our sample size is small, this high rate of resistance of Spn and Hib to co-trimoxazole is consistent with hospital-based findings from Bangladesh reported by Saha et al. (31,32). Other studies conducted in this region have also reported similar patterns (33,34). A multi-country study in the Asia-Pacific region reported co-trimoxazole resistance rates of 18% and 15% in Spn and Hib isolates obtained from blood samples from patients with community-acquired ALRIs (35). Resistance to co-trimoxazole has been increasing in Bangladesh, and it has been attributed to long-term and widespread use of this drug (31,36). The antimicrobial resistance patterns differ widely between countries in Asia and Europe, highlighting the importance of local data in guiding the choice of treatment of community-acquired respiratory tract infections (37). The high rate of resistance against co-trimoxazole raises concerns about the current recommendation that this drug be used as the first-line drug for the treatment of ALRI.

Qualitative research is needed to understand the reasons for the low use of hospital care for ALRIs in neonates and to design strategies to improve care-seeking in this vulnerable age-group. The seasonality needs to be further evaluated with environmental factors that might influence these variations. This study presumably under-estimated the burden of diseases due to Hib and Spn. Since Hib and Spn conjugate vaccines have the potential to significantly reduce child mortality, evaluation of alternative approaches to ascertain the burden of diseases due to Hib and Spn are needed. Future aetiologic studies should systematically look for other pathogens in addition to Hib and Spn.
